# Practice-Based Evidence: Profiling the Safety of Cilostazol by Text-Mining of Clinical Notes

**DOI:** 10.1371/journal.pone.0063499

**Published:** 2013-05-23

**Authors:** Nicholas J. Leeper, Anna Bauer-Mehren, Srinivasan V. Iyer, Paea LePendu, Cliff Olson, Nigam H. Shah

**Affiliations:** 1 Divisions of Vascular Surgery and Cardiovascular Medicine, Stanford University, Stanford, California, United States of America; 2 Stanford Center for Biomedical Informatics Research, Stanford University, Stanford, California, United States of America; 3 Palo Alto Medical Foundation, Palo Alto, California, United States of America; University of Illinois-Chicago, United States of America

## Abstract

**Background:**

Peripheral arterial disease (PAD) is a growing problem with few available therapies. Cilostazol is the only FDA-approved medication with a class I indication for intermittent claudication, but carries a black box warning due to concerns for increased cardiovascular mortality. To assess the validity of this black box warning, we employed a novel text-analytics pipeline to quantify the adverse events associated with Cilostazol use in a clinical setting, including patients with congestive heart failure (CHF).

**Methods and Results:**

We analyzed the electronic medical records of 1.8 million subjects from the Stanford clinical data warehouse spanning 18 years using a novel text-mining/statistical analytics pipeline. We identified 232 PAD patients taking Cilostazol and created a control group of 1,160 PAD patients not taking this drug using 1∶5 propensity-score matching. Over a mean follow up of 4.2 years, we observed no association between Cilostazol use and any major adverse cardiovascular event including stroke (OR = 1.13, CI [0.82, 1.55]), myocardial infarction (OR = 1.00, CI [0.71, 1.39]), or death (OR = 0.86, CI [0.63, 1.18]). Cilostazol was not associated with an increase in any arrhythmic complication. We also identified a subset of CHF patients who were prescribed Cilostazol despite its black box warning, and found that it did not increase mortality in this high-risk group of patients.

**Conclusions:**

This proof of principle study shows the potential of text-analytics to mine clinical data warehouses to uncover ‘natural experiments’ such as the use of Cilostazol in CHF patients. We envision this method will have broad applications for examining difficult to test clinical hypotheses and to aid in post-marketing drug safety surveillance. Moreover, our observations argue for a prospective study to examine the validity of a drug safety warning that may be unnecessarily limiting the use of an efficacious therapy.

## Introduction

Peripheral arterial disease (PAD) is a growing problem that now accounts for every fifth dollar spent on inpatient cardiovascular care in the United States [1]. This condition affects approximately 8 million Americans, and is associated with significantly impaired long-term cardiovascular outcomes [2]. For example, PAD patients have been shown to have high rates of mortality, stroke and myocardial infarction (MI), with an equal or even greater risk of events than those subjects with a diagnosis of cerebrovascular or coronary artery disease [3]. Patients with claudication also report reduced quality of life, experience higher rates of clinical depression, and are measurably more sedentary than non-PAD patients [4]–[6].

Despite the impact of this disease, very few medical therapies are available to the patient with PAD. Indeed, Cilostazol is the only FDA-approved medication that carries a class I indication for the treatment of intermittent claudication [7]. Cilostazol is a type III phosphodiesterase inhibitor that possesses both vasodilatory and anti-platelet properties, and has been shown to improve maximal walking distance significantly compared to placebo in a series of prospective randomized clinical trials [8], [9]. Cilostazol can induce a number of minor side effects such as headache and diarrhea, but generally has been observed to be safe with regards to major cardiovascular events such as myocardial infarction, stroke and death [10], [11]. However, other phosphodiesterase inhibitors such as milrinone have been associated with increased mortality rates in patients with congestive heart failure (CHF) [12], and Cilostazol has therefore been issued a black box warning despite never having been shown to increase risk of any major clinical endpoint [12], [13]. Prior attempts to quantify this risk were underpowered and did not lead to reversal of the FDA’s risk assessment [14]. To additionally quantify the risk associated with this black box warning, we developed a novel text-analytics pipeline to examine the adverse event profile [15] of Cilostazol in a clinical setting, and also in patients with CHF.

## Methods

### Data Sources

We used clinical notes from the Stanford Translational Research Integrated Database Environment (STRIDE). For validation of our findings in the CHF subgroup we used data from the Palo Alto Medical Foundation (PAMF).

The STRIDE dataset spans 18-years’ worth of data from 1.8 million patients; it contains 19 million encounters, 35 million coded ICD9 diagnoses, and a combination of pathology, radiology, and transcription reports totaling over 11 million unstructured clinical notes.

The PAMF dataset spans 13-years’ worth of patient data from 1.2 million patients; it contains 78 million encounters, 64 million coded ICD9 diagnoses, and a combination of progress notes, pathology, radiology, and transcription reports totaling over 50 million unstructured clinical notes.

The use of these data sources has been approved by the Institutional Review Boards at Stanford and PAMF.

### Data Collection and Processing

**Figure 1:**
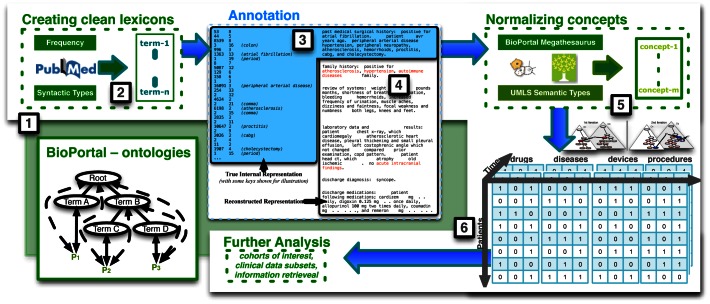
Generation of the patient–feature matrix. (1) The workflow downloads ∼5.6 M strings from the 22 clinically relevant ontologies as well as trigger terms from NegEx and ConText for negation detection. (2) It uses term frequency and syntactic type information (e.g., predominant noun phrases) from MedLine to prune the set of strings into a clean lexicon, and (3) then applies the lexicon directly against the textual clinical notes using exact string matching. (4) The workflow furthermore uses NegEx and ConText rules to filter negated terms and terms within family history contexts. (5) Next, UMLS and BioPortal mappings and semantic type information are used to normalize terms into concepts, which are furthermore grouped into the semantic groups “drug”, “disease”, “device”, or “procedure”. (6) Finally, the annotations of the clinical notes are used to construct the patient–feature matrix, where each row of the matrix represents a patient and the columns are the clinical concepts annotated in the patients clinical notes; here the time stamps of the clinical notes induce a temporal ordering of the annotations over the entire patient–feature matrix.

We processed the unstructured clinical notes as described in Figure 1 and by LePendu *et al*. [16]. In brief, we used an optimized version of the NCBO Annotator [17], [18] with a set of 22 clinically relevant ontologies. We removed ambiguous terms using a variety of statistical and manual filters [19]–[22], and flagged negated terms as well as terms attributed to family history [23], [24]. We normalized all drugs to their ingredients using RxNorm, such that the terms “pletal” and “cilostazol” are both normalized to the ingredient Cilostazol. We normalized remaining terms to clinical concepts and aggregated the concepts according to hierarchical relationships, e.g., patients with acute myocardial infarction are also counted as persons with myocardial infarction. Finally, we ordered the set of all concepts for each note based on the time at which the note was recorded. As a result, for every patient, we have sets of concepts spaced apart in time based on the clinical notes they were mentioned in, comprising the patient-feature matrix (see Figure 1).

We recognize drug exposure and clinical conditions based on the temporally ordered concept mentions. We validated the accuracy using a manually annotated gold standard corpus (from the 2008 i2b2 Obesity Challenge [25]). This corpus is manually annotated by two annotators for 16 conditions and was designed to evaluate the ability of NLP systems to identify a condition present for a patient based on textual notes. On average, we achieved 98% specificity for recognizing disease conditions with a precision of 90%. In particular, for PAD we have 98% specificity (with 83% precision) and for CHF 95% specificity (with 92% precision). We trade sensitivity for ensuring high specificity and precision; and sensitivity is around 73%. However, given the large dataset we begin with, we are still able to identify large enough cohorts for the study. Drug recognition is done in a similar manner using strings from RxNORM and an independent study at the University of Pittsburgh, which examined the annotations on 1960 clinical notes manually [26], estimated over 84% sensitivity and 84% specificity for recognizing drugs.

### Study Covariates and Outcome Variables

We defined several covariates for propensity score matching and several outcome variables for comparison. Each variable is composed of a set of concepts, and each concept contains several terms. For example, the variable “myocardial infarction” is composed of 18 different concepts, including C0027051 (myocardial infarction), C0340324 (silent myocardial infarction) and C0155626 (acute myocardial infarction), etc. (see Material S1). Each of these concepts can be further decomposed into the terms, which are actually mentioned in the clinical notes. For example, the terms “heart attack” and “myocardial infarction” both count as mentions of the concept C0027051 (myocardial infarction). The list of concepts and terms defining the covariates as well as the outcome variables used in this study was manually curated and can be found in the Material S1.

We defined an index time point of treatment for all patients, and grouped all annotations into two groups: concepts associated with clinical events that happened before treatment (which can therefore be used for matching patients) and concepts associated with events that happened after the treatment (and can therefore be interpreted as outcomes). We scanned the annotations of each patient for the occurrence of concepts before and after the index time point to create a binary matrix; where for each patient we set the variable to 1 or 0 indicating that the concepts had been mentioned in the clinical notes or not. We extracted the demographic variables age, gender and race, and used a cross-reference of the STRIDE data with the social security index (SSDI) to define the outcome variable “death (SSDI)”.

### Study Period and Study Groups

We extracted data from our annotations for all patients with PAD, as defined by mention of the peripheral artery disease terms listed in Table 1. To allow a detailed analysis of multiple clinical endpoints, we excluded patients having less than one year’s worth of data after their first PAD mention to ensure sufficient clinical follow up data for each patient. For the Cilostazol study group, we selected those PAD patients who had a Cilostazol mention after or at the same time as their first PAD mention. We then used 1∶5 propensity score matching to define a control group.

**Table 1:** Peripheral artery disease definition.

Concept	Concept unique identifier (UMLS)
Peripheral arterial diseases	C1704436
Peripheral vascular diseases	C0085096
Peripheral arterial occlusive disease	C1306889
Intermittent claudication	C0021775
Claudication (finding)	C1456822

To summarize, patients in the Cilostazol study group met the following criteria: (i) they had to have a diagnosis of PAD as defined by mention of the PAD-related terms listed above, (ii) the first PAD mention had to be before or at the same time as the Cilostazol mention, (iii) the patients were required to have at least one year worth of data after their first PAD mention. The control group similarly carried a diagnosis of PAD, had no mention of Cilostazol, and was matched to the Cilostazol group by propensity score matching based on expert selected variables.

### Congestive Heart Failure Study Subgroup

In addition to the total PAD group, we also extracted patients who had a mention of CHF in their clinical notes before the first mention of Cilostazol. The electronic records of these subjects containing the CHF annotation were manually reviewed to confirm the clinical diagnosis of CHF and to ensure the correctness of the temporal ordering. We then used 1∶5 propensity score matching to construct a control group from all other PAD patients who also had a history of CHF, but no Cilostazol prescription.

### Propensity Score Matching and Statistical Methods

We used propensity score matching to construct control groups. For this purpose, we first fit a propensity score model using logistic regression where the treatment assignment (Cilostazol vs. no Cilostazol) was regressed on the 18 covariates marked in Table 2, including the demographic variables, age at first PAD mention, gender and race, as well as several co-morbidities and co-prescriptions. We then used the Matching package for R [27] to perform 1∶5 propensity score matching without replacement and to check balance in the variables between the Cilostazol and control groups. We analyzed the success of the matching–whether covariate values were balanced across the two groups after matching–by examining for significant differences in means for continuous variables and significant differences in percentages for indicator variables using a p-value significance level of 0.05. To account for the matched nature of the data, we then used conditional logistic regression [28] of the Survival package for R [29] to compute odds ratios and 95% confidence intervals for several outcome variables. The same analysis was performed for the patients with a history of CHF. Furthermore, we performed standard multivariate logistic regression to compute odds ratios which: 1) compare the Cilostazol group with all other unmatched PAD patients, 2) adjust for confounding by including several covariates, as well as the propensity scores themselves in the regression model (see Material S2).

**Table 2:** Balance in variables before and after propensity score matching in STRIDE.

Variable	Before Matching			After PSM matching	
	Cilostazol group (n = 232)	Unmatched PAD patients (n = 5525)	p-value	Matched control group (n = 1160)	p-value
**Demographics**					
Age (at indication onset), mean (sd)*	71.20 (10.98)	70.41 (12.47)	0.30	71.43 (10.87)	0.81
**Gender (female), n (%)** *	**37.07**	**45.96**	**<0.01**	**36.03**	**0.82**
Race, n (%)*					
Asian	8.62	7.40	0.52	8.10	0.84
Black	2.59	3.71	0.30	2.76	0.91
**Native American**	**0.00**	**0.24**	**<0.001**	**0.00**	**1.00**
Unknown	24.14	26.12	0.50	24.66	0.90
White	64.22	62.26	0.54	63.97	0.95
**Comorbidities**					
Congestive heart failure, n (%)*	18.53	21.96	0.19	19.22	0.84
Diabetes, n (%)	25.00	19.55	0.06	25.69	0.86
**Dyslipidemias, n (%)** *	**57.33**	**47.22**	**<0.01**	**58.62**	**0.77**
**Hypertension, n (%)** *	**74.41**	**68.07**	**0.04**	**75.17**	**0.78**
Renal failure, n (%)*	9.05	7.78	0.51	8.88	0.95
**Co-prescriptions**					
**Statins, n (%)** *	**62.50**	**48.25**	**<0.001**	**63.79**	**0.76**
**Beta blocking agents, n (%)**	**50.86**	**43.44**	**0.03**	**50.09**	**0.86**
**ACE inhibitors, plain, n (%)** *	**61.21**	**53.17**	**0.02**	**61.64**	**0.92**
Antiplatelet drugs, n (%)					
**Aspirin, n (%)** *	**68.10**	**58.32**	**<0.01**	**69.14**	**0.80**
**Clopidogrel, n (%)** *	**31.03**	**14.46**	**<0.001**	**28.19**	**0.44**
Warfarin, n (%)*	13.36	17.20	0.10	11.64	0.57
**Antiarryhtmics, n (%)** *	**25.86**	**36.09**	**<0.001**	**25.78**	**0.98**
**Diabetes drugs, n (%)**	**29.74**	**23.71**	**0.05**	**29.05**	**0.87**
**History of**					
Arrhythmias, n (%)*	32.76	31.78	0.76	33.71	0.82
Tachycardia, n (%)	21.55	21.77	0.94	20.78	0.84
Atrial fibrillation, n (%)	12.93	14.10	0.61	13.28	0.91
Ventricular tachycardia, n (%)	3.02	2.44	0.62	2.84	0.60
Ventricular fibrillation, n (%)	0.86	0.81	0.94	0.60	0.75
Conduction disease and/or bradyarrythmia, n (%)	12.93	12.24	0.76	14.91	0.53
MACE, n (%)* †	29.74	30.24	0.87	29.48	0.95
Myocardial infarction, n (%)	18.10	15.87	0.39	16.98	0.75
Stroke, n (%)	18.97	18.30	0.80	16.98	0.57
Defibrillation event, n (%)	2.59	3.49	0.40	3.19	0.70
Cardiac arrest, n (%)	1.29	1.05	0.75	1.21	0.93
Sudden cardiac death, n (%)	0.43	0.58	0.78	0.43	1.00
**MALE, n (%)** †	**79.74**	**48.56**	**<0.001**	**80.43**	**0.76**
**Revascularization, n (%)** *	**75.43**	**42.01**	**<0.001**	**76.29**	**0.72**
**Bypass, n (%)** *	**35.35**	**17.03**	**<0.001**	**33.19**	**0.57**
**Angioplasty, n (%)** *	**28.02**	**11.66**	**<0.001**	**25.00**	**0.39**
Amputation, n (%)*	6.73	4.87	0.16	7.07	0.92

Variables that differ statistically significantly (p-value <0.05) are bold. Propensity score matching removes any imbalance in all variables.

* covariates included in the propensity score model,

† pooled variables combining all variables listed below.

## Results

In the current paper, we describe a study performed using free-text clinical notes from the clinical data warehouse at Stanford. Our text-processing pipeline converts clinical notes from a patient’s medical record into a patient-feature matrix for data mining as described in the Methods. In order to study the outcomes in patients with PAD taking Cilostazol, we examined for differences in several clinical outcomes comparing patients taking Cilostazol with a matched control group. As described in the methods, we defined an index time point (the time point at which treatment for PAD started) and scanned the patient’s annotations for occurrence of the variables before and after that time point. We then used variables mentioned before the index time point for propensity score matching and variables mentioned after that time point as outcome variables. We analyzed outcomes between the 232 patients on Cilostazol in STRIDE, and their matched controls by comparing for significant differences in major adverse cardiovascular events (MACE), major adverse limb events (MALE), and symptoms for arrhythmias. We also examined a small cohort of patients with congestive heart failure who were prescribed Cilostazol and validated our findings for the CHF subgroup in an independent dataset.

### Propensity Score Matching

In total, there were 11,435 PAD patients in STRIDE. Amongst the entire cohort, there was no difference in mortality (OR = 1.08 CI [0.86, 1.35]) comparing 340 Cilostazol patients with the other 11,095 PAD patients, as assessed by query of the SSDI. In order to carry out a more detailed analysis of multiple clinical endpoints such as MACE and MALE, we restricted our study set of 11,435 PAD patients to the 5,757 PAD patients with at least one year of clinical follow up, as described in the methods. For this reduced study set, we had on average more than 8 years’ worth of data spanning the index time point of treatment for each patient In this group, we identified 232 PAD patients taking Cilostazol and compared them to the other 5,525 PAD patients in the STRIDE database. Table 2 summarizes the prevalence of several clinical variables in the Cilostazol study group and the unmatched PAD control patients. On average, the Cilostazol patients are older, are more likely male, have more comorbidities, are prescribed more medications and have had more major adverse limb events than PAD patients not taking Cilostazol (p-value <0.05 for each condition); hence on average Cilostazol patients are sicker than the other PAD patients. After using propensity score matching, we were able to identify a cohort of 1160 controls (1∶5 matching) that were fully balanced for all 18 clinical variables (see Table 2). This group was used to compare all subsequent clinical outcomes. In total, 5,892 patient-years of data were available for the subjects studied compared to 2136 patient-years in [14].

### Outcomes in PAD Patients Taking Cilostazol

#### Differences in claudication symptoms

We first quantified the frequency with which subjects in each group reported improvement or resolution of claudication symptoms over time. We were able to ‘re-discover’ that Cilostazol use was associated with a significant reduction in symptomatology [30]–defined by mentions of phrases such as “no claudication”, “no complaints of claudication”, or “no sign of claudication” after assignment to the Cilostazol group (OR = 2.35, CI [1.75, 3.14]–thus providing a positive control for our approach.

Another example that such text-mining approaches don’t always result in negative findings, is given by our recently published study, in which we used similar techniques to detect adverse drug reactions from the clinical notes and achieved 80.4% AUC on a gold standard of positive and negative drug-adverse event associations as well as detected 6 out of 9 recalls in the past decade including the association between Vioxx and Myocardial infarction [15], [16].

#### Differences in major adverse cardiovascular events (MACE)

**Figure 2:**
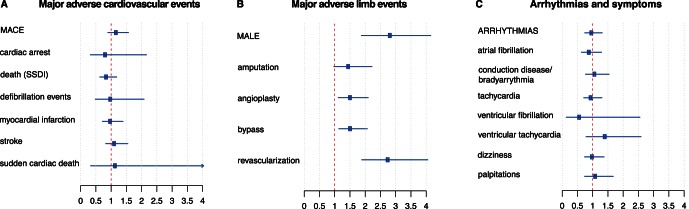
Outcomes in PAD patients taking Cilostazol compared to the matched control group. Odds ratios and 95% confidence intervals are plotted; upper limits of the confidence intervals are clipped at 4. There are no statistically significant differences in major adverse cardiovascular events (A), there is an increased risk for several major adverse limb events (B), and there are no differences for arrhythmias and arrhythmic symptoms (C). MACE and MALE are pooled variables combining all other variables listed below.

To assess the impact of Cilostazol therapy on major clinical outcomes, we then computed odds ratios for several major adverse cardiovascular events (MACE), including myocardial infarction, stroke, cardiac arrest, sudden cardiac death and defibrillation events. Compared to the entire unmatched PAD cohort, those prescribed Cilostazol had slightly higher rates of MACE (crude OR = 1.37, CI [1.05, 1.79]). However, after matching on potential confounders, Cilostazol was not associated with any major cardiovascular endpoint including death (OR = 0.86, CI [0.63, 1.18]), MI (OR = 1.00, CI [0.71, 1.39]), or stroke (OR = 1.13, CI [0.82, 1.55]) in the matched cohort (see Figure 2A). Similar results were obtained adjusting the crude odds ratios for different potential confounders (see Material S2).

#### Differences in major adverse Limb events (MALE)

To assess the impact of Cilostazol therapy on PAD-specific outcomes, we next compared major adverse limb events (MALE) such as amputation and lower extremity revascularization. As expected, the Cilostazol group had much more advanced PAD than the unmatched control PAD group, with significantly higher rates of MALE (crude OR = 6.26, CI [4.30, 9.13]) and each PAD-specific endpoint (see Material S2). Compared to the matched control group, the difference in odds ratios between the groups reduced, but still remained significantly different for MALE (OR = 2.84, CI [1.87, 4.29]), amputation (OR = 1.47, CI [0.97, 2.22]), bypass (OR = 1.53, CI [1.14, 2.07]) and revascularization (OR = 2.77, CI [1.89, 4.05]) (see Figure 2B). Again, similar results were obtained using different ways to adjust for confounders (see Material S2).

#### Differences in arrhythmias and arrhythmic symptoms

Despite the concern that Cilostazol may increase malignant arrhythmias, we did not observe any statistically significant differences between the Cilostazol and control PAD patients (either before or after matching) with respect to cardiac arrhythmias, nor typical arrhythmia symptoms (see Figure 2C) and Material S2).

### Outcomes in PAD Patients with CHF Taking Cilostazol

**Figure 3:**
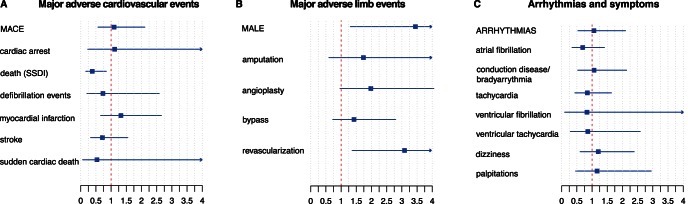
Outcome analysis in the CHF subgroup comparing patients with a history of CHF and taking Cilostazol to a matched control of CHF patients not taking Cilostazol. Odds ratios and 95% confidence intervals are plotted; upper limits of the confidence intervals are clipped at 4. There is no statistically increased risk for any major adverse cardiovascular events (A), there is an increased odds ratio for several major adverse limb events (B), and there are no differences for arrhythmias and arrhythmic symptoms (C). MACE and MALE are pooled variables combining all other variables listed below.

We identified several patients who had an annotation of CHF before the first mention of Cilostazol. After manually reviewing their medical records, we confirmed that 43 patients with a diagnosis of CHF were subsequently prescribed Cilostazol for PAD. We used these patients to comprise a CHF study subgroup. Again, we observed an imbalance in several variables including gender, several co-prescriptions and history of revascularization events. Using propensity score matching, we extracted a control group of 215 PAD patients who also had a history of CHF but were not prescribed Cilostazol, and then compared both groups with respect to different outcomes. Matching removed pre-existing imbalance in the covariates (see Material S3). Importantly, Cilostazol use was not associated with an increase in any major adverse cardiovascular event amongst heart failure patients. Similarly, no increase in arrhythmia, arrhythmic symptoms, or sudden cardiac death was observed in this subgroup analysis (see Figure 3). We again observed slightly increased odds ratios for major adverse limb events, in particular revascularization events, confirming that the PAD of the Cilostazol patients was more advanced.

We also extracted data for 96 PAD patients with a history of CHF who were prescribed Cilostazol from an independent data source at PAMF. We manually validated the CHF subgroup similarly as done for the STRIDE dataset. Using propensity score matching we constructed a fully balanced matched control group of 480 patients (for balance analysis see Material S4), and analyzed differences in clinical outcomes between the two groups using the same methods as for STRIDE data. We observed the same trend as seen for the STRIDE data in Figure 3 (see Table 3).

**Table 3:** Outcomes in the CHF-subgroup in the PAMF dataset comparing Cilostazol patients with their matched controls.

Major adverse cardiovascular events		Major adverse limb events		Arrhythmias and symptoms	
MACE*	1.38 [0.85, 2.25]	MALE*	0.89 [0.54, 1.46]	ARRHYTHMIAS	1.05 [0.65, 1.70]
Cardiac arrest	1.07 [0.44, 2.64]	Amputation	1.63 [0.87, 3.03]	Atrial fibrillation	0.90 [0.57, 1.42]
Defibrillation events	0.82 [0.34, 2.01]	Angioplasty	1.43 [0.87, 2.36]	Conduction disease/bradyarrhythmia	1.34 [0.85, 2.09]
Myocardial infarction	1.42 [0.90, 2.26]	Bypass	1.18 [0.73, 1.93]	Tachycardia	1.15 [0.73, 1.82]
Stroke	0.91 [0.57, 1.43]	Revascularization	0.92 [0.54, 1.46]	Ventricular fibrillation	1.67 [0.45, 6.16]
Sudden cardiac death	0.38 [0.05, 2.94]			Ventricular tachycardia	1.32 [0.56, 3.13]
				Dizziness	0.99 [0.63, 1.55]
				Palpitations	1.18 [0.73, 1.90]

* pooled variables combining all variables listed below.

## Discussion

In this study, we employed a novel analytical approach to conduct the equivalent of a phase IV safety surveillance study on an efficacious, yet potentially dangerous FDA-approved drug. By querying the clinical medical records of over 1.8 million patients with our pipeline, we were able to identify a large cohort of PAD subjects that were matched with the exception of exposure to Cilostazol, the agent of interest in this study. Using this approach, we did not observe any difference in mortality comparing the Cilostazol patients to all other unmatched PAD patients. We furthermore observed no association between Cilostazol and any major adverse cardiovascular event including stroke, myocardial infarction or death in a reduced fully matched study set, which is in good agreement with earlier studies [31]. We also identified a subset of CHF patients who were prescribed Cilostazol, and interestingly found that it did not appear to increase mortality in this theoretically high-risk group of patients. This proof of principle study shows the potential of data-mining methods to query unstructured data in clinical data warehouses to answer important, but difficult to address clinical questions [32]. Moreover, it argues for a prospective study to examine the validity of an unproven FDA-issued black box warning that likely limits the broad application of a clinically effective therapy.

In many situations, clinical hypotheses often go untested due to ethical concerns around presumed benefit. Examples include the use of PVC-suppressing antiarrythmics post MI or hormone replacement in menopausal women, each of which was found to promote, not prevent risk when formally tested [33], [34]. Similarly, clinical trials often do not study the most complicated patients due to concerns over the impact of comorbidities, and clinicians often have little data to guide therapy for the sickest patients. We argue that in the era of electronic medical records, it is possible to harness the knowledge embedded in clinical data warehouses to inform therapy decisions [32] as well as perform phase IV surveillance [15], [16], [35]. The informatics approaches employed in the current study allow for uncovering ‘natural experiments’ that would otherwise be difficult to perform–generating practice-based evidence.

By looking at large enough sample sets, it is possible to identify patients of interest who have been exposed to a given treatment approach, compare them to patients who are otherwise indistinguishable, and observe their clinical outcomes for significant differences. Because this work is performed with data from a ‘real world’ clinical setting, patients who would have been excluded from most clinical trials are also examined, such as the patients with recognized CHF who were prescribed Cilostazol. Given Cilostazol’s black box warning, it is difficult to imagine a scenario where these patients would have been enrolled into a trial that was supported by a pharmaceutical company and endorsed by an academic Institutional Review Board. While our findings do not prove that Cilostazol is safe in heart failure patients, they help make the case for a prospective study in this cohort.

Because the full medical record can be queried, this approach also offers the benefit of allowing a wide spectrum of endpoints to be assessed. Also, at-risk and other understudied subgroups such as children, the elderly, minorities, pregnant women and those with multiple comorbidities could be studied with this approach. In the current study, we focused heavily on potential arrhythmic complications given the high incidence of palpitations reported in the original Cilostazol studies. Importantly, no increase in arrhythmia was observed and there was no increase in total mortality or sudden cardiac death – endpoints, which would have been detected by cross-referencing with the Social Security Death Index.

This study has several potential limitations that warrant discussion. Although our annotation pipeline has been shown to have a specificity of 98% for recognizing diseases, we could have missed comorbidities due to false negatives from lower sensitivity (73%). However, these errors should be equally distributed across case and control groups. We performed standard propensity score matching in order to reduce potential bias introduced by imbalance in the covariates; however matching may not have been complete. For example, we did not have access to the subjects’ ankle-brachial indices, and therefore could not quantitate the severity of each patient’s peripheral stenosis at baseline. Indeed, we observed that the Cilostazol group had higher rates of MALE than control subjects. While we cannot exclude the possibility that Cilostazol promotes the progression of PAD, we view this as an unlikely possibility given the multiple published randomized, placebo-controlled trials demonstrating efficacy of Cilostazol [10], [11]. Rather, we suspect that the groups were not completely matched for PAD severity at baseline, given that Cilostazol is generally prescribed to subjects with lifestyle-limiting claudication [3], [36]–[38]. As a result, the Cilostazol group may have had higher-grade ischemic lesions, which necessitated the observed increase in peripheral interventions and MALE. However, if an unmeasured residual imbalance was present, it would bolster the interpretation that Cilostazol is likely safe from a cardiovascular mortality perspective, in that the treatment group presumably had more advanced atherosclerosis, yet had no increase in arrhythmia or cardiovascular events when taking the drug. Moreover, we applied different models including a variety of additional potential confounders and the results did not change (for details see Material S2). Finally, the outcome measures may not have captured events occurring outside of the hospital or that led to hospitalizations in other institutions. However, we note that the endpoint of death was captured for all patients via cross-referencing with the Social Security Death Index data, giving confidence in our conclusions about survival. Also, our ‘re-discovery’ that Cilostazol reduces claudication complaints provides a ‘positive control’ to illustrate the potential of our approach for detecting subjective clinical endpoints.

In conclusion, we used an informatics approach to examine the side-effect profile of Cilostazol and to indirectly assess the validity of a black box warning that was originally issued over theoretical concerns. We find that the feared complications of malignant arrhythmia and sudden death were not observed in association with the drug in the cohort examined. We used our analytics approach to discover and examine a ‘natural experiment’ in a subset of patients that would be difficult to enroll in a clinical trial and found that Cilostazol had no untoward effect on survival amongst heart failure patients. This result supports the argument for a prospective randomized trial in CHF patients, which need not be considered unsafe or unethical.

We believe that similar Phase IV monitoring could be executed for other drugs without a proven safety record to identify sequelae not recognized at the time of FDA review. We expect that such data-mining driven surveillance approaches will have broad applicability to the field of pharmaceutical safety and will become a key aspect of Phase IV post-marketing surveillance, particularly for patient groups not likely to be studied in randomized clinical trials.

## Supporting Information

Material S1 **Variable definitions:** The concepts and terms defining the variables used in this study. The table also includes the frequency of each concept/term in the PAD cohort.
(XLSX)

Material S2 **Outcomes analysis using multivariate logistic regression – STRIDE dataset.**
(PDF)

Material S3 **Balance in variables before and after propensity score matching – CHF subgroup in STRIDE.**
(PDF)

Material S4 **Balance in variables before and after propensity score matching – CHF subgroup in PAMF.**
(PDF)
