# Supplementary material for: Practice-Based Evidence: Profiling the Safety of Cilostazol by Text-Mining of Clinical Notes
Source: PLoS One. 2013 May 23;8(5):e63499. doi: 10.1371/journal.pone.0063499 (PMC3662653; doi:10.1371/journal.pone.0063499)
Supplement: Material S2 — Outcomes analysis using multivariate logistic regression – STRIDE dataset. (PDF) [file pone.0063499.s002.pdf]

Table S2: Outcomes analysis using multivariate logistic regression - STRIDE dataset

Clinical outcome	OR [95% CI] (crude)	OR [95% CI]* (psm-adjusted)	OR [95% CI] † (adjusted for age, gender, history of MACE, history of MALE)	OR [95% CI] ‡ (adjusted for age, gender, co-morbidities, co-prescriptions)
**Major adverse cardiovascular events**				
MACE	1.37 [1.05, 1.79]	1.25 [0.95, 1.63]	1.31 [0.99, 1.74]	1.24 [0.93, 1.65]
cardiac arrest	0.94 [0.38, 2.31]	0.75 [0.26, 1.70]	0.80 [0.28, 1.80]	0.81 [0.28, 1.83]
defibrillation events	0.89 [0.45, 1.76]	0.90 [0.42, 1.69]	0.81 [0.38, 1.52]	0.81 [0.38, 1.53]
myocardial infarction	1.27 [0.93, 1.73]	1.05 [0.76, 1.44]	1.11 [0.99, 1.74]	1.02 [0.73, 1.40]
stroke	1.23 [0.92, 1.65]	1.20 [0.88, 1.61]	1.24 [0.90, 1.67]	1.19 [0.87, 1.61]
sudden cardiac death	1.56 [0.48, 5.05]	1.30 [0.30, 3.60]	1.47 [0.35, 4.15]	1.03 [0.24, 2.97]
death (SSDI)	1.00 [0.74, 1.34]	0.89 [0.63, 1.18]	0.84 [0.61, 1.13]	0.87 [0.63, 1.18]
**Major adverse limb events**				
MALE	6.26 [4.30, 9.13]	3.33 [2.25, 5.07]	4.25 [2.84, 6.54]	3.71 [2.48, 5.72]
amputation	2.21 [1.51, 3.24]	1.62 [1.07, 2.38]	1.62 [1.08, 2.37]	1.68 [1.10. 2.49]
angioplasty	2.60 [1.96, 3.43]	1.52 [2.23, 2.06]	1.83 [1.37, 2.44]	1.70 [1.26, 2.26]
bypass	2.69 [2.07, 3.51]	1.57 [1.17, 2.10]	1.81 [1.36, 2.39]	1.71 [1.29, 2.27]
revascularization	5.76 [4.10, 8.08]	3.21 [2.24, 4.67]	3.96 [2.76, 5.79]	3.54 [2.47, 5.19]
**Arrhythmias and symptoms**				
ARRHYTHMIAS	1.11 [0.85, 1.44]	1.09 [0.83, 1.43]	1.03 [0.78, 1.36]	1.03 [0.78, 1.36]
atrial fibrillation	0.91 [0.65, 1.26]	0.98 [0.69, 1.37]	0.85 [0.60, 1.19]	0.91 [0.63, 1.27]
conduction disease/ bradyarrhythmia	1.21 [0.88, 1.68]	1.13 [0.81, 1.57]	1.13 [0.80, 1.57]	1.08 [0.76, 1.50]
tachycardia	0.98 [0.74, 1.31]	1.03 [0.77, 1.37]	0.93 [0.69, 1.24]	0.96 [0.71, 1.29]
ventricular fibrillation	0.77 [0.19, 3.15]	0.74 [0.12, 2.46]	0.61 [0.10, 2.00]	0.59 [0.10, 1.96]
ventricular tachycardia	1.56 [0.91, 2.68]	1.45 [0.80, 2.44]	1.34 [0.74, 2.25]	1.33 [0.73, 2.25]
dizziness	1.05 [0.78, 1.40]	1.05 [0.77, 1.40]	1.07 [0.79, 1.43]	1.03 [0.76, 1.38]
palpitations	1.15 [0.78, 1.68]	1.24 [0.83, 1.81]	1.26 [0.84, 1.84]	1.23 [0.82, 1.80]

* Odds ratios adjusted using the propensity score as covariate in the multivariate logistic regression
† Odds ratios adjusted for age, gender and history of MACE and MALE events
‡ Odds ratios adjusted for age, gender, history of MACE, MALE, dyslipidemias, hypertension, statins use, ace inhibitors use and diabetes drugs use
