# Supplementary material for: Practice-Based Evidence: Profiling the Safety of Cilostazol by Text-Mining of Clinical Notes
Source: PLoS One. 2013 May 23;8(5):e63499. doi: 10.1371/journal.pone.0063499 (PMC3662653; doi:10.1371/journal.pone.0063499)
Supplement: Material S3 — Balance in variables before and after propensity score matching – CHF subgroup in STRIDE. (PDF) [file pone.0063499.s003.pdf]

Table S3: Balance in variables before and after propensity score matching – CHF subgroup in STRIDE

Variable	Before Matching			After PSM matching	
	Cilostazol group (n= 43)	Unmatched PAD patients (n= 1213)	p-value	Matched control group (n= 215)	p-value
**Demographics**					
Age (at indication onset), mean (sd) *	72.58 (10.03)	72.15 (12.45)	0.79	73.01 (10.30)	0.85
**Gender (female), n (%)***	**27.91**	**42.95**	**0.04**	**29.30**	**0.88**
Race, n (%)*					
Asian	4.65	8.66	0.24	3.26	0.75
Black	2.33	5.60	0.18	0.93	0.61
**Native American**	**0.41**	**0.00**	**0.03**	**0.00**	**1.00**
Unknown	24.14	35.81	0.34	35.81	0.75
White	58.14	59.60	0.85	59.01	0.93
**Comorbidities**					
Diabetes, n (%)*	34.88	33.64	0.87	32.56	0.82
Dyslipidemias, n (%)	69.77	58.94	0.14	73.49	0.68
Hypertension, n (%)	88.37	85.74	0.60	87.91	0.95
Renal failure, n (%)	9.05	7.78	0.51	8.88	0.95
**Co-prescriptions**					
**Statins, n (%)***	**81.40**	**61.50**	**<0.001**	**81.40**	**1.00**
**Beta blocking agents, n (%)**	**88.37**	**68.67**	**<0.001**	**80.93**	**0.34**
ACE inhibitors, plain, n (%)*	70.07	75.10	0.54	82.33	0.70
Antiplatelet drugs, n (%)					
Aspirin, n (%)*	90.70	75.93	0.54	82.33	0.94
**Clopidogrel, n (%)***	**48.84**	**23.58**	**<0.001**	**46.98**	**0.83**
Warfarin, n (%)*	23.26	30.59	0.28	22.33	0.92
Antiarryhtmics, n (%)*	53.49	52.43	0.89	55.35	0.86
Diabetes drugs, n (%)*	48.84	40.64	0.30	47.44	0.90
**History of**					
Arrhythmias, n (%)*	65.12	60.92	0.58	68.84	0.70
Tachycardia, n (%)	53.49	46.91	0.41	50.23	0.77
Atrial fibrillation, n (%)	30.23	30.17	0.99	30.70	0.97
Ventricular tachycardia, n (%)	9.30	6.68	0.57	8.37	0.89
Ventricular fibrillation, n (%)	4.65	2.97	0.61	4.65	1.00
Conduction disease and/or bradyarrythmia, n (%)	30.23	26.55	0.61	33.02	0.78
MACE, n (%)*†	51.16	53.09	0.81	49.77	0.90
Myocardial infarction, n (%)	41.86	32.40	0.23	32.56	0.36
Stroke, n (%)*	25.58	28.19	0.70	27.44	0.85
Defibrillation event, n (%)*	9.30	9.15	0.97	8.84	0.94
**Cardiac arrest, n (%)***	**0.00**	**2.80**	**<0.001**	**0.00**	**1.00**
Sudden cardiac death, n (%)	2.33	1.73	0.80	2.33	1.00
**MALE, n (%)†**	**83.72**	**61.83**	**< 0.001**	**83.26**	**0.95**
**Revascularization, n (%)***	**79.07**	**52.68**	**<0.001**	**76.29**	**0.91**
Bypass, n (%)*	39.53	26.13	0.09	33.19	0.86
Angioplasty, n (%)*	25.58	14.43	0.11	25.00	1.00
Amputation, n (%)*	4.65	6.92	0.50	7.07	0.92

* covariates included in the propensity score model, † pooled variables combining all variables listed below
