# Supplementary material for: Practice-Based Evidence: Profiling the Safety of Cilostazol by Text-Mining of Clinical Notes
Source: PLoS One. 2013 May 23;8(5):e63499. doi: 10.1371/journal.pone.0063499 (PMC3662653; doi:10.1371/journal.pone.0063499)
Supplement: Material S4 — Balance in variables before and after propensity score matching – CHF subgroup in PAMF. (PDF) [file pone.0063499.s004.pdf]

Table S4: Balance in variables before and after propensity score matching – CHF subgroup in PAMF

Variable	Before Matching			After PSM matching	
	Cilostazol group (n= 93)	Unmatched PAD patients (n= 3378)	p-value	Matched control group (n= 465)	p-value
**Demographics**					
**Age (at indication onset), mean (sd) ***	**74.76 (9.05)**	**62.05 (17.43)**	**<0.001**	**75.02 (8.85)**	**0.91**
Gender (female), n (%)*	55.91	59.27	0.52	56.13	0.98
**Comorbidities**					
**Diabetes, n (%)**	**45.16**	**23.62**	**<0.001**	**47.74**	**0.72**
**Dyslipidemias, n (%)***	**87.10**	**57.79**	**<0.001**	**87.10**	**1.00**
**Hypertension, n (%)***	**95.70**	**63.23**	**<0.001**	**94.62**	**0.69**
**Renal failure, n (%)***	**23.66**	**12.37**	**0.01**	**25.16**	**0.81**
**Co-prescriptions**					
**Statins, n (%)***	**82.80**	**44.85**	**<0.001**	**83.87**	**0.81**
**Beta blocking agents, n (%)**	**78.49**	**41.06**	**<0.001**	**76.56**	**0.73**
**ACE inhibitors, plain, n (%)***	**91.40**	**58.08**	**<0.001**	**90.54**	**0.81**
Antiplatelet drugs, n (%)					
**Aspirin, n (%)***	**87.10**	**51.81**	**<0.001**	**88.60**	**0.74**
**Clopidogrel, n (%)**	**48.84**	**12.55**	**<0.001**	**36.13**	**0.49**
Warfarin, n (%)	24.73	17.20	0.10	35.27	0.11
**Antiarryhtmics, n (%)***	**62.37**	**44.58**	**<0.001**	**64.95**	**0.71**
**Diabetes drugs, n (%)**	**48.39**	**23.24**	**<0.001**	**49.46**	**0.88**
**History of**					
**Arrhythmias, n (%)***	**59.14**	**35.02**	**<0.001**	**59.14**	**1.00**
**Tachycardia, n (%)**	**40.86**	**23.36**	**0.001**	**43.23**	**0.75**
Atrial fibrillation, n (%)	24.73	15.96	0.06	32.47	0.24
Ventricular tachycardia, n (%)	3.23	1.89	0.48	4.73	0.57
Ventricular fibrillation, n (%)	2.15	0.77	0.37	1.72	0.82
**Conduction disease and/or bradyarrythmia, n (%)***	**27.96**	**16.02**	**0.01**	**30.75**	**0.67**
**MACE, n (%)*†**	**62.37**	**29.54**	**<0.001**	**64.95**	**0.70**
**Myocardial infarction, n (%)***	**39.78**	**16.76**	**<0.001**	**40.00**	**0.98**
Stroke, n (%)	37.63	18.71	**<0.001**	41.08	0.64
Defibrillation event, n (%)	3.23	3.52	0.87	8.17	0.16
Cardiac arrest, n (%)	5.38	1.84	0.14	5.38	1.00
Sudden cardiac death, n (%)	1.08	0.80	0.80	1.08	1.00
**MALE, n (%)*†**	**77.42**	**24.75**	**<0.001**	**79.57**	**0.41**
**Revascularization, n (%)***	**79.07**	**52.68**	**<0.001**	**70.54**	**0.68**
**Bypass, n (%)***	**38.71**	**9.41**	**<0.001**	**34.84**	**0.48**
**Angioplasty, n (%)***	**26.88**	**3.14**	**<0.001**	**26.02**	**0.88**
Amputation, n (%)	10.75	3.14	0.02	6.67	0.33

* covariates included in the propensity score model, † pooled variables combining all variables listed below
